# Supplementary material for: Rapid high-resolution measurement of DNA replication timing by droplet digital PCR
Source: Nucleic Acids Res. 2018 Jul 9;46(19):e112. doi: 10.1093/nar/gky590 (PMC6212846; doi:10.1093/nar/gky590)
Supplement: Supplementary Data [file gky590_supplemental_files.pdf]

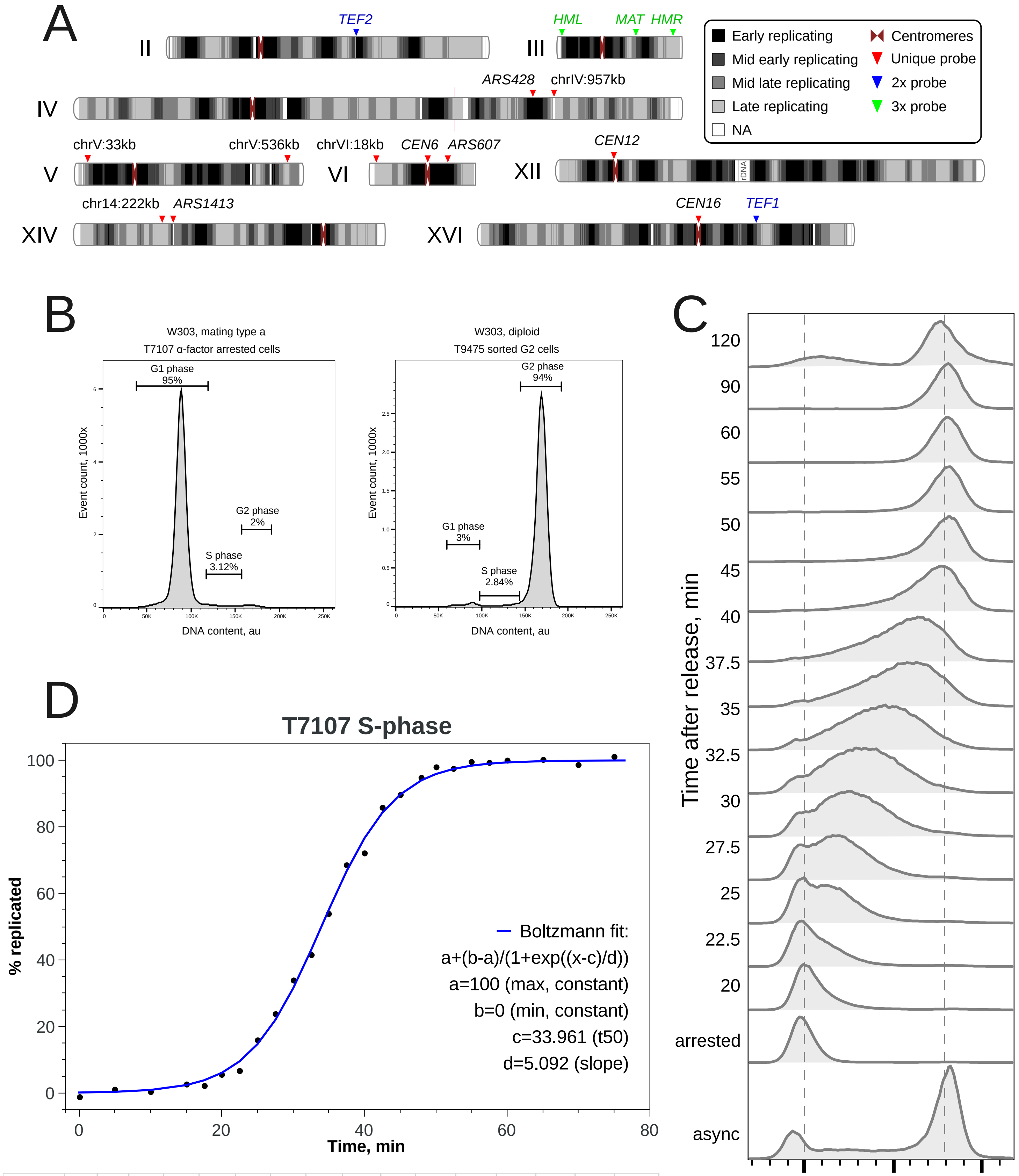

Time	0	20	22.5	25	27.5	30	32.5	35	37.5	40	42.5	45	48	50	60	90
Boltzman	0.1	6.1	9.5	14.7	21.9	31.5	42.9	55.1	66.7	76.6	84.3	89.7	94.0	95.9	99.4	100.0

Supplemental Figure 1.

A, Schematic representation of genomic location and replication timing of probes used in Figure 1. *B*, Flow cytometry profiles of G1 arrested (left) and FACS-enriched G2 (right) cells used in Figure 1. *C*, Flow cytometry profiles of cell cycle experiment used in Figures 1, 2 and 3. *D*, Sigmoidal curve fit of DNA fluorescence for the cell cycle experiment in *C*.

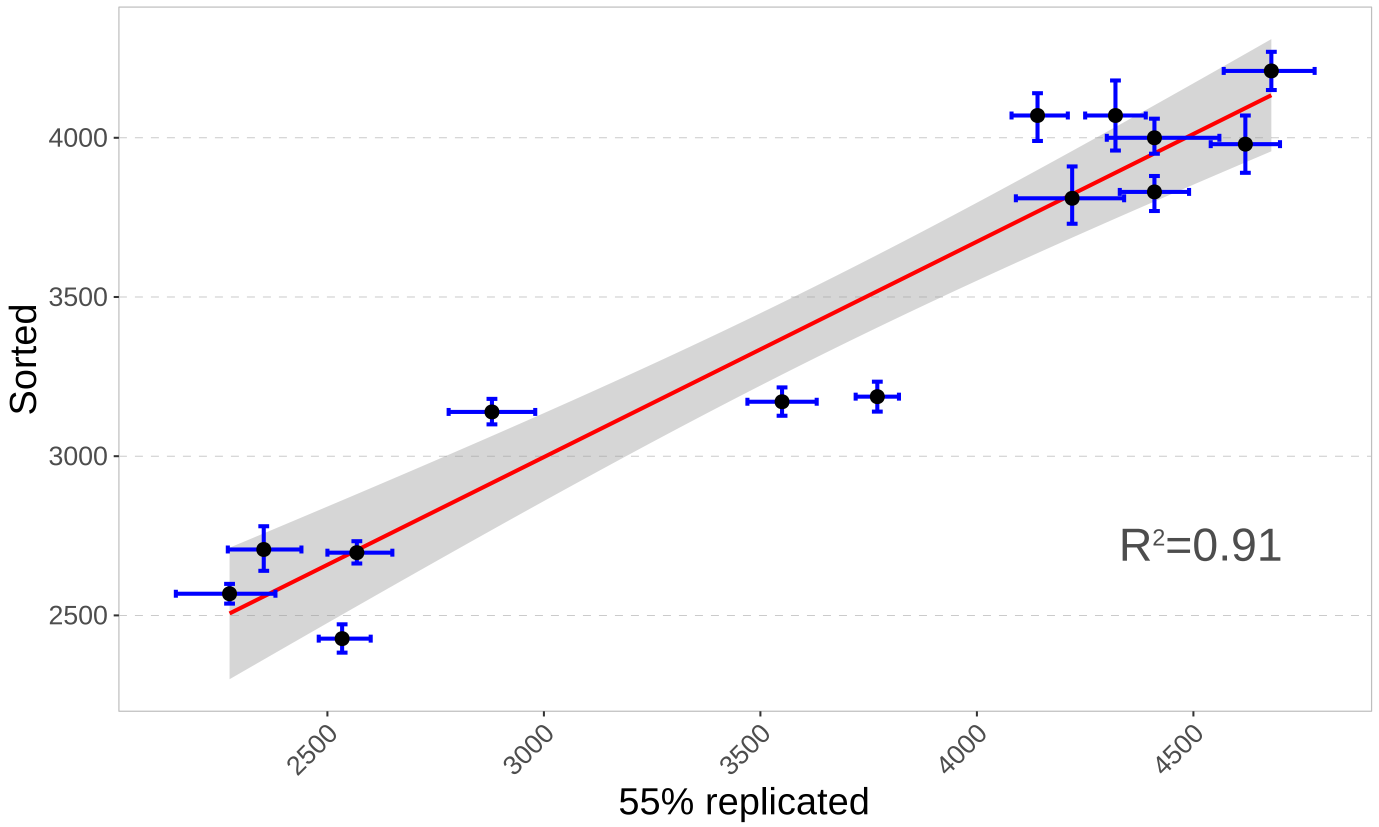

Supplemental Figure 2.

Linear fit between probe concentration from Figure 1B. Red line - the estimated fit with 0.95 CI grey band.

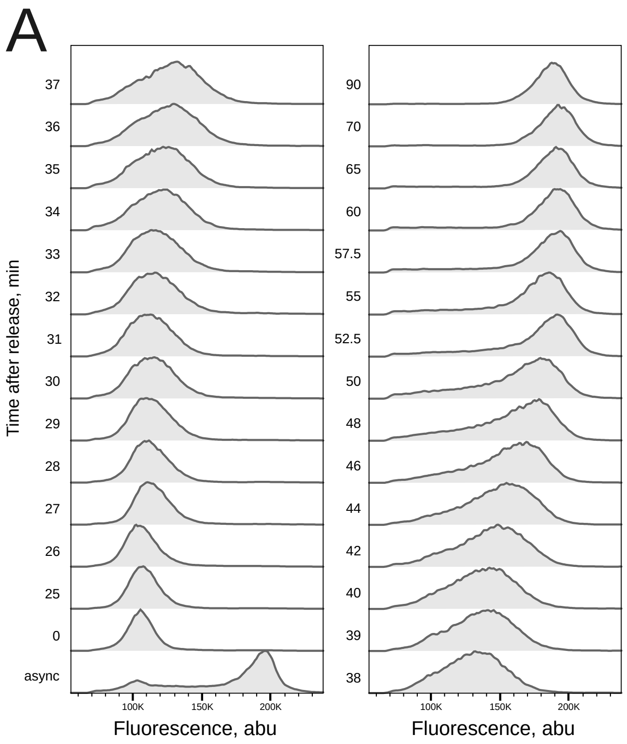

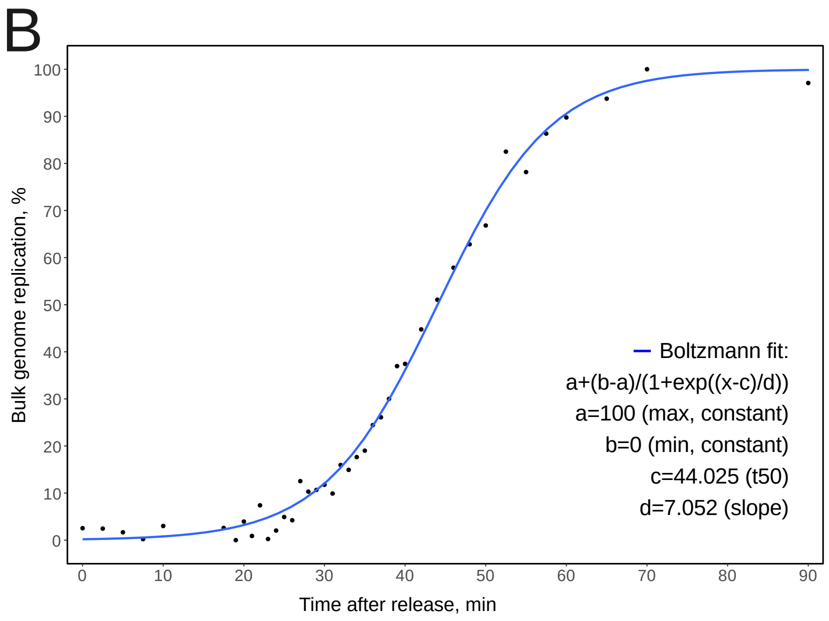

Time	0	10	15	20	25	30	35	40	42	44	46	50	55	70
Boltzman	0.2	0.8	1.6	3.2	6.3	12.0	21.8	36.1	42.9	49.9	57.0	70.0	82.6	97.6

Supplemental Figure 3.

A, Flow cytometry profiles of cell cycle experiment used in Figure 4. *B*, Sigmoidal curve fit of DNA fluorescence for the cell cycle experiment in *A*.

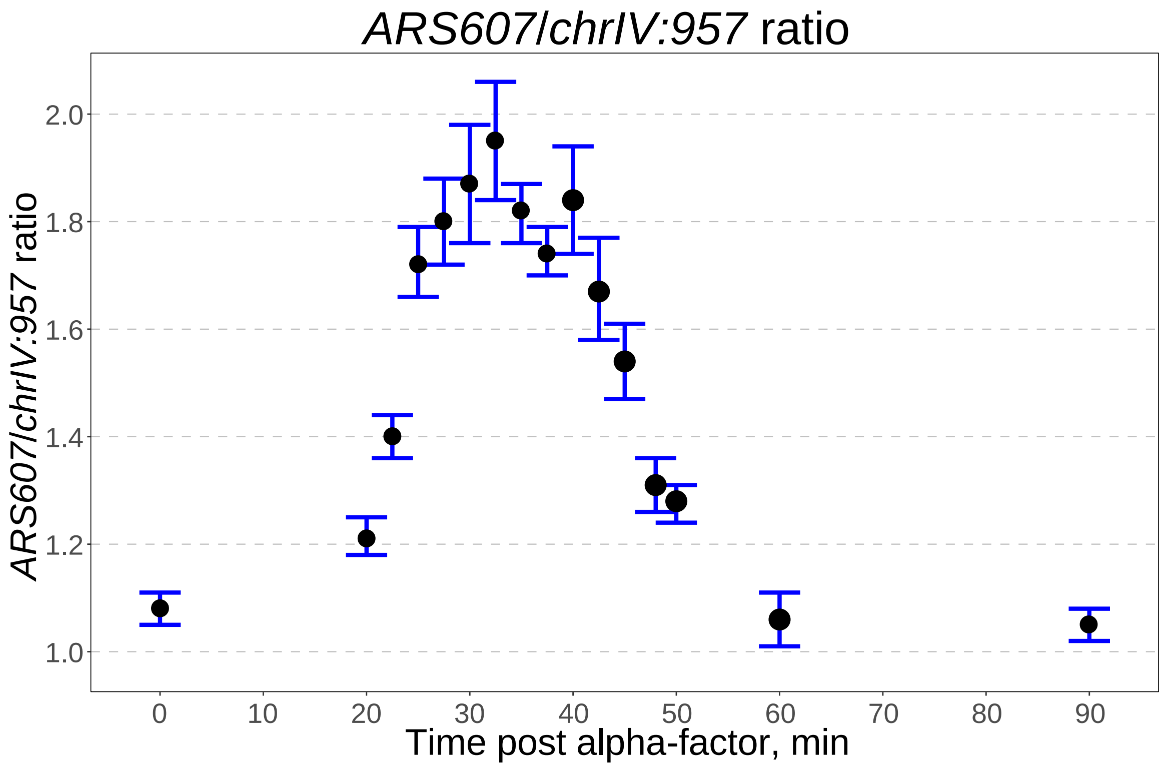

Supplemental Figure 4.

Biological replicate of experiment shown in Figure 4A. The samples are from the cell cycle experiment shown in the Supplemental Figures 1C and 1D.

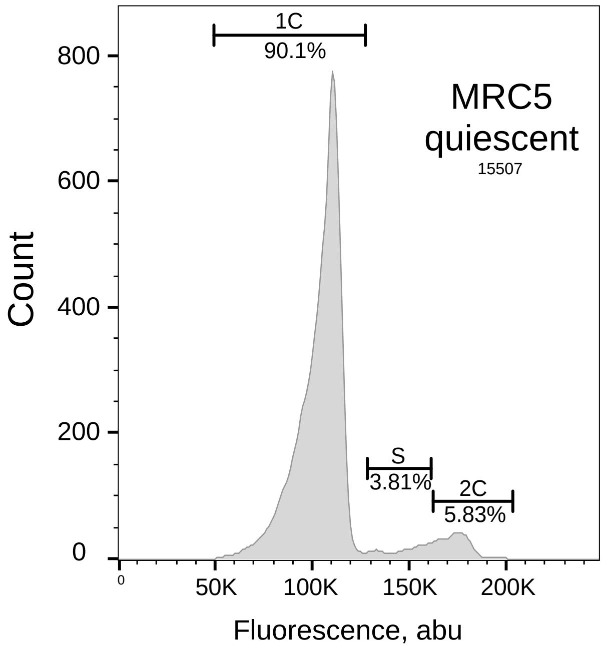

Supplemental Figure 5.

Flow cytometry profile of G1 arrested MRC-5 cells from Figure 6A.

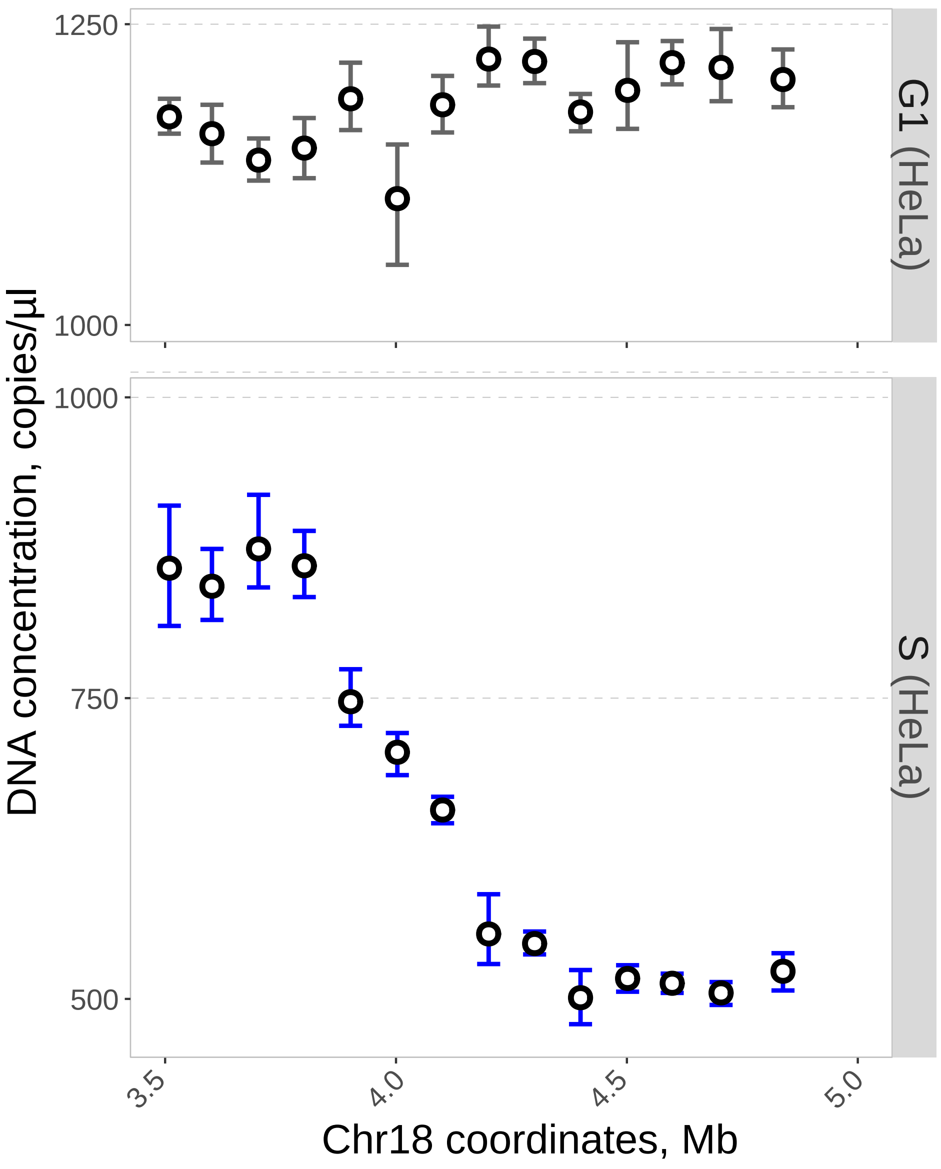

Supplemental Figure 6.

Biological replicate of the experiment presented in the Figure 6B using SYTOX Green instead of Propidium Iodide to stain DNA prior to FACS.

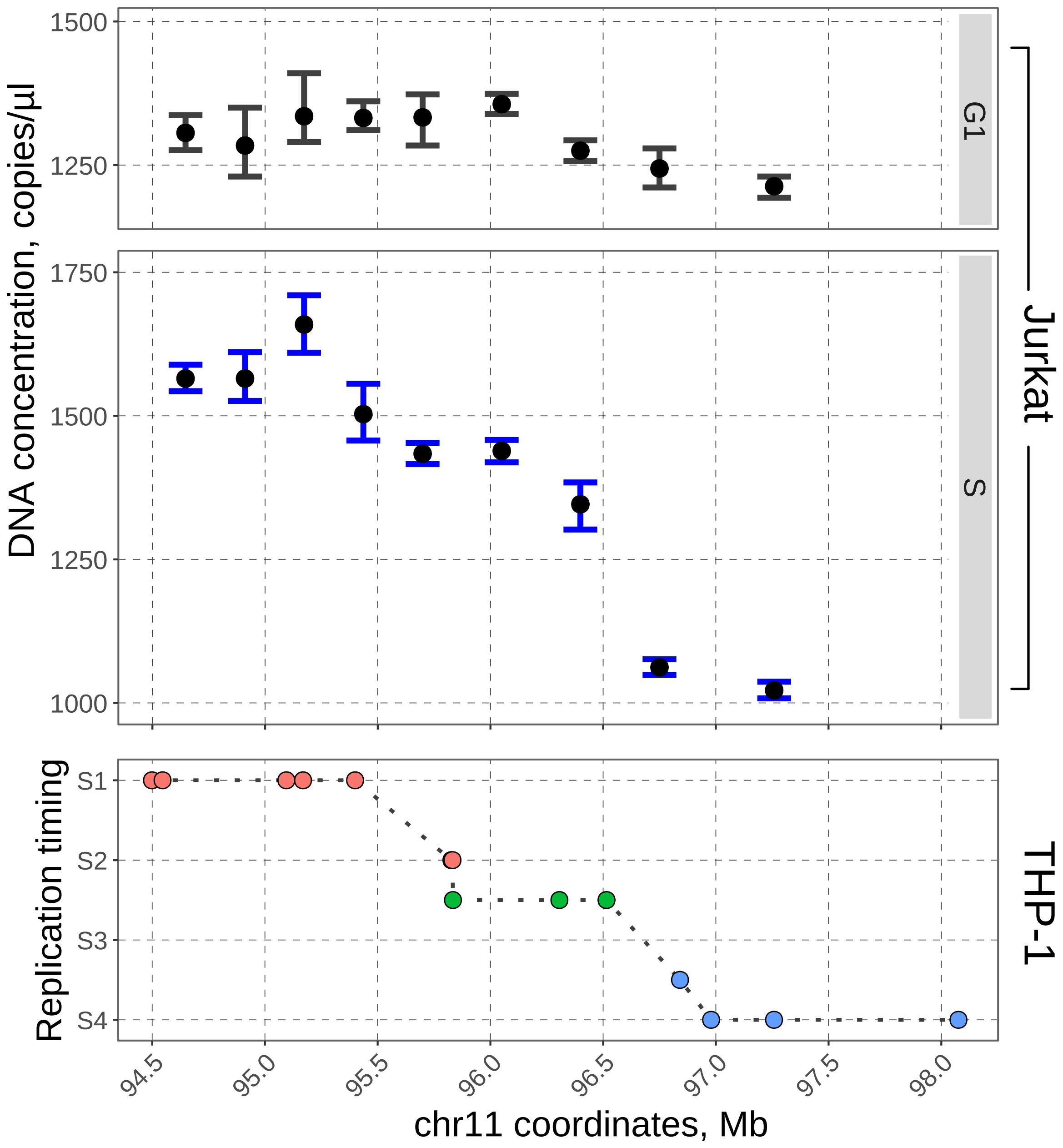

Supplemental Figure 7.

DNA concentration analysed by ddPCR across a replication timing transition zone on chromosome 11 in Jurkat cells. *Top,* ddPCR analysis of DNA from non-replicating FACS-enriched G1 cells. *Middle,* ddPCR analysis of DNA from replicating FACS-enriched S phase cells. *Bottom:* replication timing from corresponding genomic region in TMP-1 cells, retaining original colors, with red marking early replicating, green - mid-late and blue - late replicating sequences (Watanabe *et al.*, 2004).

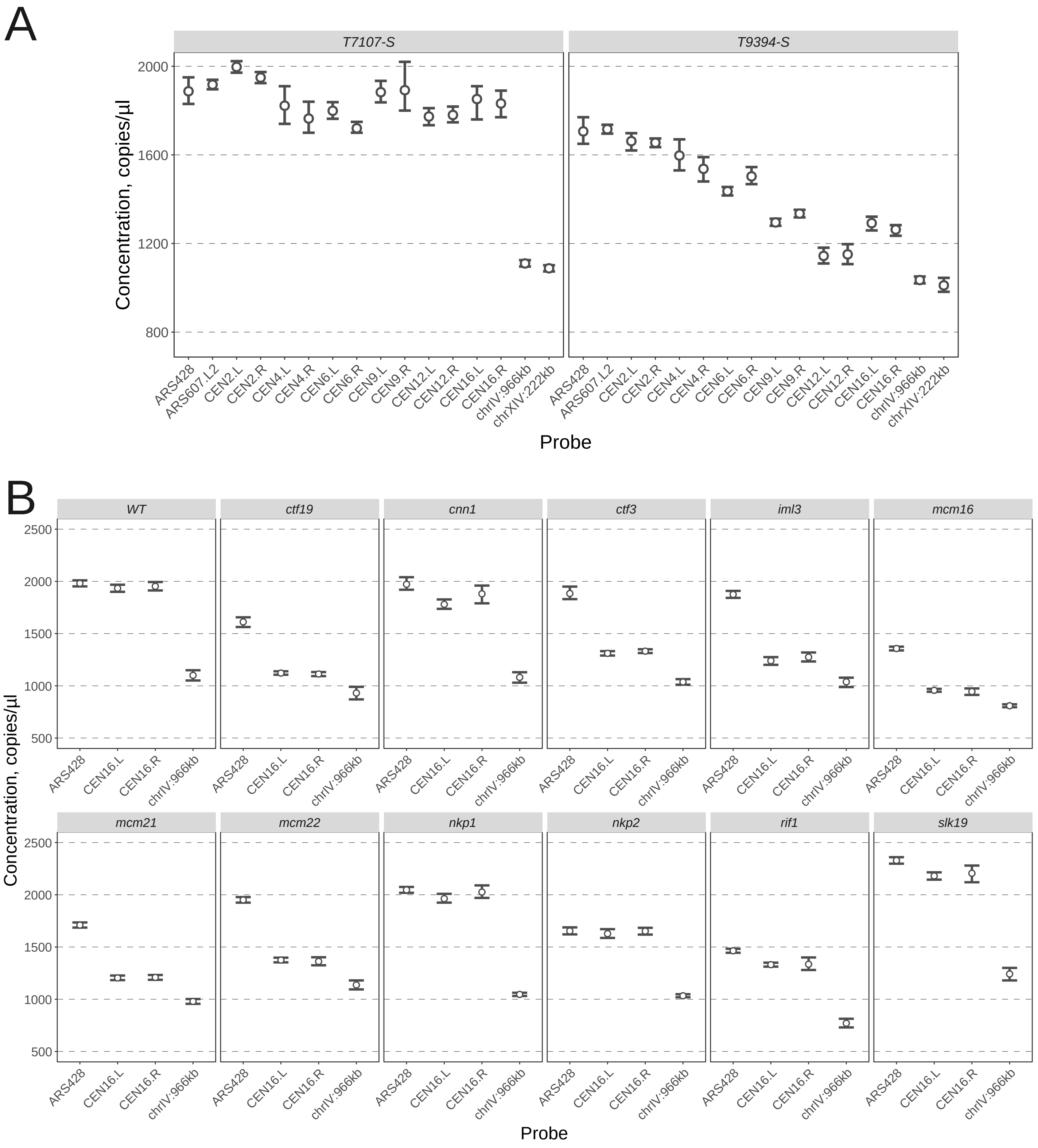

Supplemental Figure 8.

A, Probe concentrations for samples in Figure 7A. *B*, Probe concentrations for samples in Figure 7B.

A

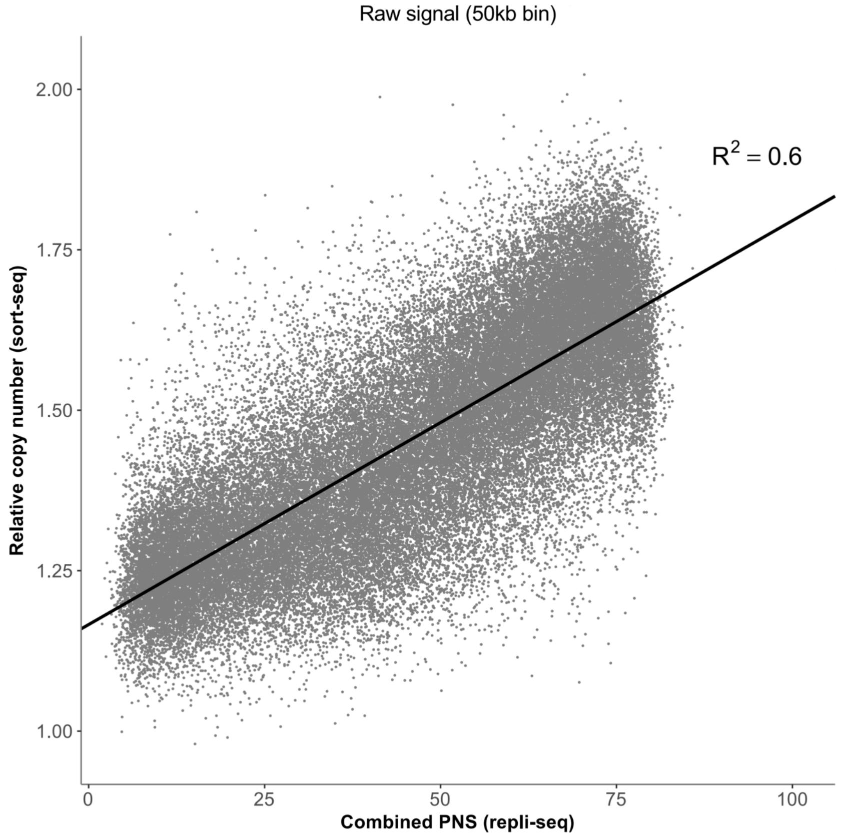

B

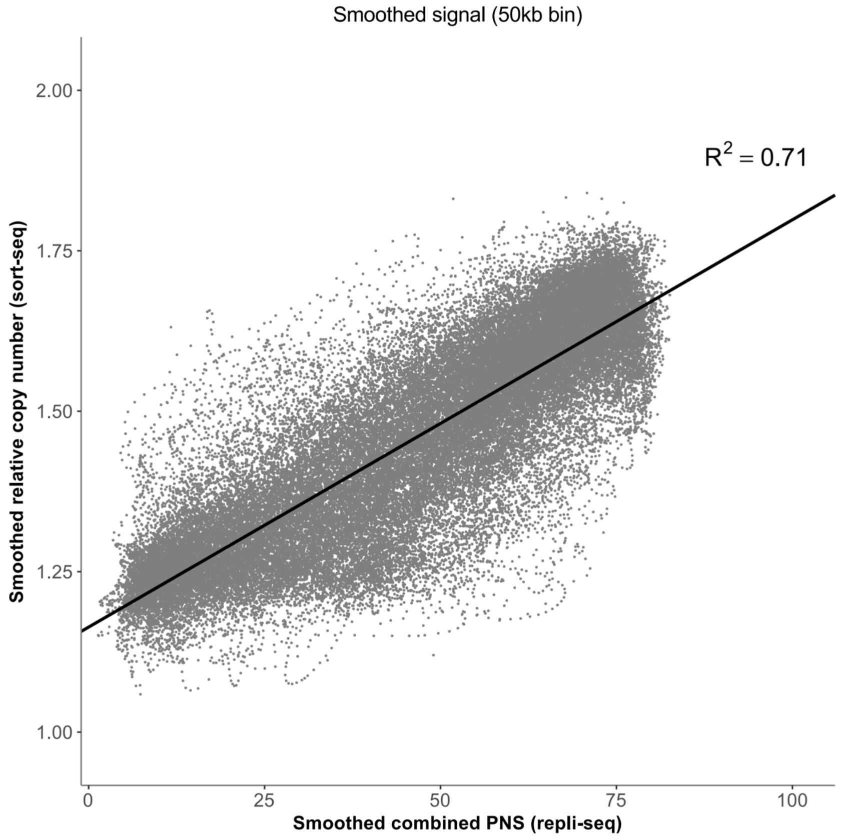

Supplemental Figure 9.

Comparison of replication values in 50 kb bins generated by sort-seq (this study) and repli-seq (Hansen *et all*., 2010). A, raw data. B, Smoothed data. The lines indicate linear fit between the two datasets. PNS - percentage-normalised signal.

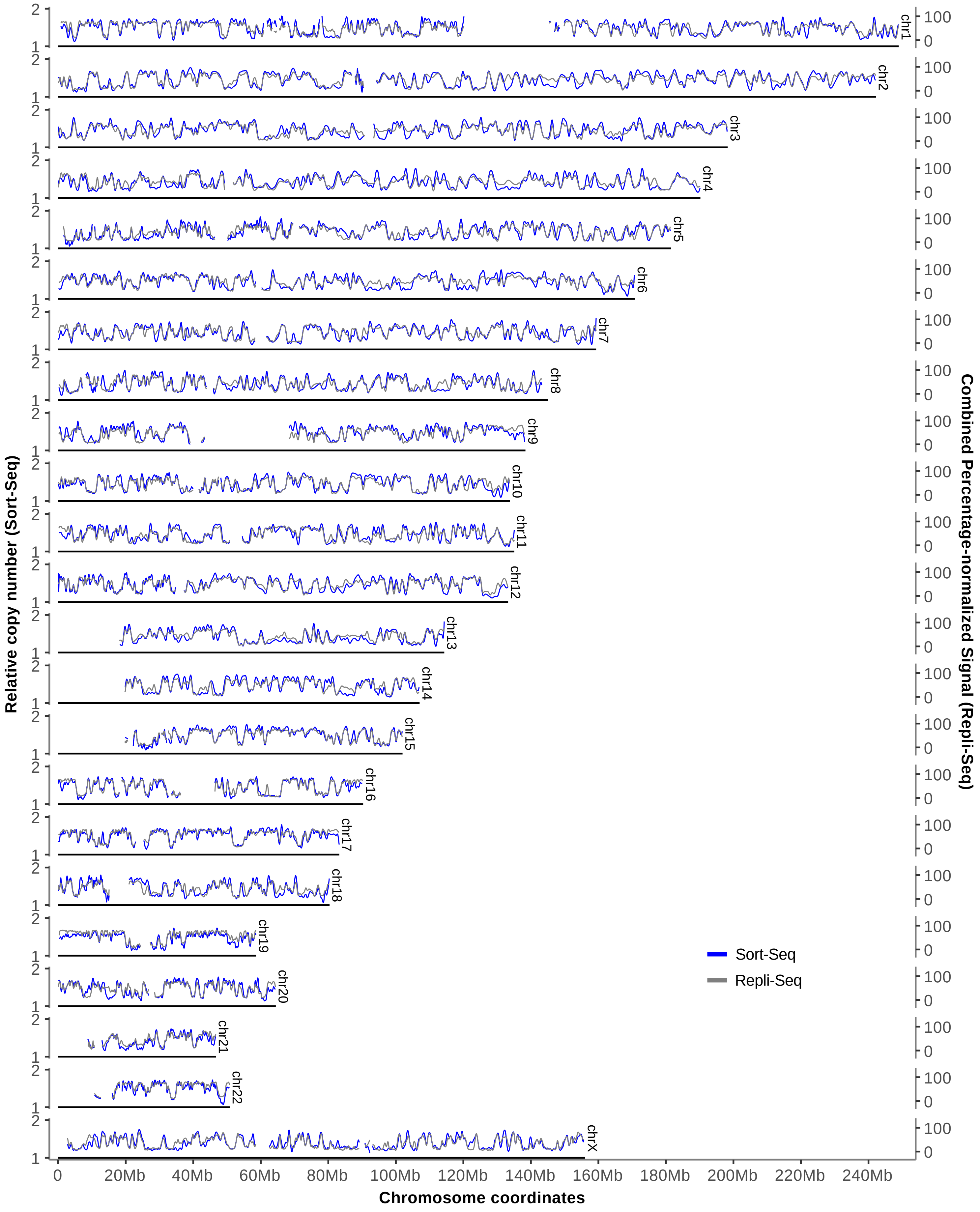

Supplemental Figure 10.

Comparison of cubic spline smoothed replication profiles generated by sort-Seq (this study) and repli-Seq (Hansen *et all*., 2010)

Strain	Genotype	Background	Source
T7107	*MATa, RAD5, BUD4, leu2, ura3, trp1, ade2, his3*	W303	T. Tanaka
T9475	*MatA/MATalpha, RAD5/RAD5, BUD4/BUD4, leu2/leu2, ura3/ura3, trp1/trp1, ade2/ade2, his3/his3*	W303	T. Tanaka
T9394	*MATa, DBF4-9myc::hphNT1, RAD5, BUD4, leu2, ura3, trp1, ade2, his3*	W303	T. Tanaka
MHY230	*MATA/MATalpha; RAD5/RAD5; ade2-1/ade2-1; his3-11,15/his3-11,15; leu2-3,112/leu2-3,112; trp1-1/trp1-1; ura3-1/ura3-1; can1-100/can1-100; Gal+/Gal+; psi+/psi+; bar1::TRP1/bar1::TRP1; cdc7-1/cdc7-1; Ars606::ars606 ACS>XhoI/ARS606; ars1021::ars1021 ACS>XhoI/ARS1021; ars731.5::ars731.5 ACS>XhoI/ARS731.5*	W303	M. Hawkins
Δctf19	*MATa, his3Δ1, leu2Δ0, met15Δ0, ura3Δ0, ctf19ΔKanMX*	S288C	Giaever et al, 2002
Δcnn1	*MATa, his3Δ1, leu2Δ0, met15Δ0, ura3Δ0, cnn1ΔKanMX*	S288C	Giaever et al, 2002
Δctf3	*MATa, his3Δ1, leu2Δ0, met15Δ0, ura3Δ0, ctf3ΔKanMX*	S288C	Giaever et al, 2002
Δiml3	*MATa, his3Δ1, leu2Δ0, met15Δ0, ura3Δ0, iml3ΔKanMX*	S288C	Giaever et al, 2002
Δmcm16	*MATa, his3Δ1, leu2Δ0, met15Δ0, ura3Δ0, mcm16ΔKanMX*	S288C	Giaever et al, 2002
Δmcm21	*MATa, his3Δ1, leu2Δ0, met15Δ0, ura3Δ0, mcm21ΔKanMX*	S288C	Giaever et al, 2002
Δmcm22	*MATa, his3Δ1, leu2Δ0, met15Δ0, ura3Δ0, mcm22ΔKanMX*	S288C	Giaever et al, 2002
Δnkp1	*MATa, his3Δ1, leu2Δ0, met15Δ0, ura3Δ0, nkp1ΔKanMX*	S288C	Giaever et al, 2002
Δnkp2	*MATa, his3Δ1, leu2Δ0, met15Δ0, ura3Δ0, nkp2ΔKanMX*	S288C	Giaever et al, 2002
Δrif1	*MATa, his3Δ1, leu2Δ0, met15Δ0, ura3Δ0, rif1ΔKanMX*	S288C	Giaever et al, 2002
Δslk19	*MATa, his3Δ1, leu2Δ0, met15Δ0, ura3Δ0, slk19ΔKanMX*	S288C	Giaever et al, 2002

Supplemental Table 1. Yeast strains used in this study.

Probe	Forward primer	Reverse primer
ARS428	TCATCTCAACGAGCATGTGC	CCTTGTATTAATAGCGACGTTGTC
chrIV:957kb	TCCCGAAAATATTACTTGCCAAAG	AAAGGGGTTACAGGGATCAAC
chrV:33kb	TCACAACCCTCTTTCACGAC	GTATCCTCGCCATTTACTCTCG
chrV:536kb	CAGACAAAACAGCAACGGAAC	CTATATGGAAGACGTTGGGAAGG
chrVI:18kb	AACGCCTTGGGCCTAATATC	AACCCACGCCTTCCTTATTC
ARS607.L2	GTGCCAGGAGTACATAGTAACG	ACTGGTAACACTTCATGGGC
ARS607.L	TTTAGCTGGGTTTATGGGAGG	TGAACTTATACTGCCGCACG
ARS607.R	TACACATTATTCGGCACAGTAGG	ACTCTTGGTAATCAAGGCTAGAAG
CEN12.L	CTAAGTTCCGTGCTACCTGTC	AGTGTTGAGTCTTGATCGTGC
CEN12.R	GACCGTTGCCTATTCTATCCTC	CCGCTCTAAATCAGGGACTAAAG
chrXIV:222kb	ATCCTGCGCGTTGACATAA	AGATTCCGTTGCTGGCTATC
ARS1413.L	TGTGAAATGCAGTCAAAAGGTAC	GCTTTAAGGCTTTTATTTTGGGATG
ARS1413.R	GTGGATCTCCTCAATCTGGTATTT	CGTAGGCGGTTATCGTGATATG
CEN16.L	CTGCCAATGATTTCAAGCCTG	CTGAACAGCGTTTTCTCCAAC
CEN16.R	GTGACCCGTATCTCAGCTATTC	CTTGTTCTCGTGCAATGCTG
CEN6.L	TTGACATACCCGACGTTGATC	GCTCTACCAGTTCTACCACATC
CEN6.R	AGTAAACGATATGCTGGAGACG	ATGCTGATGGTAGATTGTCGG
TEF1/TEF2	CAAGGGTTGGGAAAAGGAAAC	ATGGCTTGTCAGTTGGTCTAG
MAT	CGGTGTCCTCTGTAAGGTTTAG	ACTGTATTACTCAAAGAAATGCGC
chrIV:915kb	GCCACTACTAAGGCCAATTCT	GGGAAGGCTGTTCTCACTTT
chrIV:917kb	AGACGCCTTGAAGAAGAGATTAG	ATCAACACCAGCCCAGATAC
chrIV:919kb	AAGCAGATACAGAGTGTACGAATAG	GGGCCTCTTTGATGGTGTAA
chrIV:921kb	CAGCTGAGCACAATGGAAAC	GCTTCCCATTTCCAGGGATTA
chrIV:922kb	CTGCAGGGATGGGTTATTCA	ACCTGCCAAAGATACCCTTATT
chrIV:924kb	CAGATACGCGCCTCATCTATC	GTCGTCCAGATGGTCGTTATT
chrIV:926kb	GTAGCCCGTTCTTGCTTCTT	GATCGCGTGCTGACAAATTAC
chrIV:928kb	CCAGGTCACACCAACGATTTA	GCTCTCTCCAACACCCAATAAG
chrIV:931kb	TACTCGTTCGTTCCACAGTTC	CACCAGAATGCACAACTTTACC
chrIV:932kb	CCACCGCCTTATTGGCTAAA	GGGATTTCCAAGGTCAGAAGAG
chrIV:935kb	CAGTCTGCGCAGTCCTAATATC	GGCCAGGGTCAAGTTCAATA
chrIV:937kb	GGTCATCAGATGGGAGCTAAAG	TTCCCAGACTGGTTGCTTATAC
chrIV:939kb	AAGAGCTTTGACCCACTCTTAC	CTGGCCAACACCATCATTAAAC
chrIV:941kb	CTTGACACGGAATCAACAGAAC	CGAAGACTACCTCAAGGACATAC
chrIV:943kb	CGCTTCCTTGGACTGTGTTA	CCATTCTATCCAGGCACCATTA
chrIV:945kb	TGCCCTGCATTCTTCTTAGTC	CCAAAGAAGGAAGGTCGGTAAT
chrIV:947kb	GATCACCCACTCCACGATTATG	CTTGATGAATTAAAGCAGCCCTATG
chrIV:949kb	TGTCACAACCCTCCGATAGA	GGTCCAAAGAGAGCAACTATGA
chrIV:951kb	GCCGGTTTCAGTACCCTAATAC	GATTGGAGCCTTGATCCACTAA
chrIV:953kb	ACGAAGGTGGTGGTGTATTG	CTCTTCGCATAGGCCTTCTTAT
chrIV:954kb	CAAGAGCGGATTGAGACAACTA	TGGAGCTGATAGCGACAAAC
ARS737.wt	GAAAAAGTGGAGAATTGTTTGTTTATTTTAA	TAAGCGGCAGAATTGCAAATC
ARS737.delta	GAAAAAGTGGAGAATTGTTTGTTTATTTTCT	TAAGCGGCAGAATTGCAAATC
ARS1021.wt	CGGCTAATAAGGAATCTGCTAAGA	TCACAAAAGCTGTTAATTTGAAACTAATG
ARS1021.delta	CGGCTAATAAGGAATCTGCTAAGA	TCACAAAAGCTGTTAATTTGAAACTAACT
ARS606.wt	ATTGCGAAATTGCAAACGAAAA	ACGCGTAAGGACAGATCATAAA
ARS606.delta	ATTGCGAAATTGCAAACGAACT	ACGCGTAAGGACAGATCATAAA
chrIV:966kb	ACGGCGTAATGGATCAGAAATA	CTGGCTCACCAGAATCTTCAT

Supplemental Table 2. Yeast-specific primers used in this study.

Probe	Forward primer	Reverse primer
KAL1	GAGAGCTCAGTTGAGGGTCTTG	GCTATAGTGGATGAAGACCAGACC
XIST	GGAGGCAAGATGGATGATAGC	GATGATTCTTACTGCCTCCCG
SRY	GGTACTCTGCAGCGAAGTG	GTCCTACAGCTTTGTCCAGTG
PKRY	GCTTAAGGTCACACCTTAGGG	CCACACTGTCCTTCCAAACTC
TOP1	GCAACCCTCTGCCTCTTT	GTAACAGCCTAAGTTCGCATTTG
PLCXD1	GTGGTCGGAGGACATCTTGG	GTGAATGAACCGCTTTATCGC
MYC1	GGTGGAAGGTATCCAATCCAG	GCATTGCCACGTATACTTGG
chr18:4.6Mb	TCGTCTTCGTCCAAGGCCAG	TCTCCAGGTGCACCCTCAAC
CLDN22	CCTGGACTGTTTGAGAATTGGAG	GAACCGTCTTGTGGGCAAC
ORM1	GATAAGTGTGAGCCACTGGAG	CCAAGTCTCTGTCCTGATCC
chr18:3.509Mb	ACCGTCAGGAAAGAGCTGTG	TTTCAGCACAGTGCCTAGGG
chr18:3.601Mb	CCAACCCCTCCTTTCCCATC	AGGCTGGTCTCAAATTCCCG
chr18:3.702Mb	AAGAGAGGCAGCAACAGACC	GCTCCCCTGTCTCTCAAACC
chr18:3.801Mb	CAAATCCCGACTGTGACACTTCTAG	AAGCACTTCCTCTGTGCCAG
chr18:3.901Mb	TCTCAGTTGGCCAACAGACC	CTACTGTGCTGGGAAGTGGG
chr18:4.003Mb	GAGAACCATGACCCCTCGTG	ATTCCATGGCACCTTCCCTG
chr18:4.101Mb	ACGTGGCTTCCTCACTAAGC	GGACTCCTCCATTCACCAGC
chr18:4.200Mb	GGGATCTTGTAGGCCTTCGAC	CAAAGCATACATCTGACAAGGGAC
chr18:4.300Mb	CCACCCTATGTACAGATACACCTAC	CCAGTAGAGCTCAGAATCAAGTC
chr18:4.399Mb	TAAGCAGCAACCTGACTCGG	TCAAGAGTGGTGCAGGTGTC
chr18:4.501Mb	GAGCTGGGAGATAGACGCAC	CCCTGGGTGGTATATGTGGC
chr18:4.598Mb	TCATGCCTTCTCAACCCACC	TGCCTCACCATGTGACCTTC
chr18:4.704Mb	CTGTCTTCCTGTGCCTCTCC	CCCTTGGGACATACTGGAGC
chr18:4.838Mb	GATGTGTAGGGCGAGGTACG	TGGGAGAAGGAATGCTGCTG
chr11:95.173Mb	TCAGGTCAAATGCCGAGTTATG	CACTTCTAGGTTCATGTCAATCTACT
chr11:95.699Mb	TTTCCAGCACGCCAGATT	AGCTGCCCTCAACAACAATA
chr11:96.750Mb	GCATCATACTGAGCAGCCTAAA	GGGCTATCTCTGCTATCAAACC
chr11:97.259Mb	CCATTCAAGGAAGGATTGTTTGT	GGACCATGAGCCCTTCTAATC
chr11:94.647Mb	GCATTCCAGATGCAGGGAATA	TAGCCTCATCTTGCTCCATTTC
chr11:94.911Mb	GCAGAGCCCGTGTTACTAAA	GCAAAGGGTATCAGGGAGAAA
chr11:95.436Mb	CATCCATCCTGATGACCTGTTT	TAGACCGGAGCTGTTCCTATT
chr11:96.050Mb	CAACCCAGTAAGGAAGGGTAAA	CCAGGGATGTCAGTTTCCTTAG
chr11:96.399Mb	CCCTAGCCTGGCAATGTAAA	GGCTTCCTCCTATCCGTACTA

Supplemental Table 3. Human-specific primers used in this study.

Supplemental methods.

ddPCR.

DNA extraction for ddPCR. Unbiased representation of genomic DNA is crucial for any copy number-based method. We recommend using phenol/chloroform extraction as it is an organism-independent method that has been extensively used in the generation of whole genome replication timing profiles. It is important not to denature the DNA (e.g. by heating above 80°C) to avoid potential separation of single strands into different droplets since this would double the apparent concentration when read by ddPCR (1). DNA purity is less of a concern for ddPCR than for qPCR, and we normally get good results when using fresh genomic DNA preparations even with suboptimal 260/280 ratios. However, contaminants may contribute to degradation of DNA during long-term storage. We recommend storing DNA long-term as an alcohol-precipitated pellet in ethanol at -20°C and avoiding freeze-thawing cycles of DNA solutions.

Estimation of DNA concentration prior to ddPCR. Depending on DNA purity, absorbance-based methods may not be an option when estimating DNA concentration. We routinely use Qubit dsDNA HS assay with satisfactory results.

Amount of DNA per reaction. There is an optimal copy number range for the ddPCR reaction that results in the smallest errors coming from subsampling and partitioning. The minimum error is at around 1.6 copy per droplet, which translates into 1600 copies per microlitre of the reaction. To convert genome copies into mass, a simple formula can be used:

> size of the genome in bp * average molecular weight of a single polymerised base * 1 dalton weight
>
> i.e. genome size * 618 * 1.66×10^{-24},
>
> or genome size * 1.026×10^{-21}.

For *S. cerevisiae*, the theoretical mass of a single haploid nuclear genome is 12.16×10^{6} * 1.026×10^{-21}, or ~12.48×10^{-15} g, or 12.48 fg. Therefore, for the minimal error in ddPCR, the reaction should contain $1600 \times 12.48 \times 10^{-15}$ g, or ~20 pg, of nuclear genome per microlitre. However, total DNA preparations also contain various amounts of mitochondrial DNA. To account for this and for errors in the estimation of DNA concentration, we normally aim for ~25 pg/µL, or 500 pg/reaction, when using *S.cerevisiae* total DNA.

Mammalian cells have larger genomes and, therefore, for optimal copy number in ddPCR reactions, more DNA is required. However, double stranded DNA is inhibitory for PCR (2), thus introducing additional variability to the ddPCR results. To account for this, for human samples we have used ~25 ng/reaction of genomic DNA instead of theoretically optimal 118 ng/reaction.

DNA fragmentation. For good partitioning, genomic DNA should be fragmented prior to the ddPCR reaction. We routinely use restriction enzymes, such as *EcoRI* or *BamHI*, in order to fragment genomic DNA. Amplicons must not contain recognition sites of the restriction enzyme of choice.

Primer design and concentration. In general, primer design considerations are the same as for qPCR. Where primer binding location is not constrained, we use the IDT PrimerQuest Tool for qPCR with default parameters to design primers. Where location of a single primer is constrained (typically for SNP detection), we use the IDT PrimerQuest Tool to design a matching primer using the Sequencing primer option. In this case, only amplicon size is considered and is set to 70-200 bp. Where neither primers position is constrained, only annealing temperature is considered, with a target range of 53-57°C, as determined by the nearest-neighbor method (3). Primer concentration in the ddPCR reaction determines the separation of positive from negative droplets. We routinely use 250 nM final for each primer with good results.

Reaction preparation. The reaction should be prepared in PCR vessels prior to droplet generation. We assemble 11 µL of EvaGreen Master Mix, 9 µL of fragmented total DNA and 2 µL of primer mix. As ddPCR quantification is much less affected by the reaction efficiency than qPCR, we normally prepare master mixes containing EvaGreen and template DNA, aliquot them to single reactions and add primer mixes later. This reduces pipetting errors between samples containing the same template DNA. The reaction must be well mixed prior to droplet generation.

Droplet generation. We use a multichannel pipette to transfer 20 µL of the mixed reaction to the DG8 cartridge. Droplets are generated by the QX200 Droplet Generator. Slight variations in the reaction volumes transferred at this step will only affect the total number of droplets per reaction while having no effect on the template concentration. After droplet generation is complete, we recommend using wide orifice tips to transfer the droplets into the twin.tec PCR plates (Eppendorf).

Cycling conditions. 95°C for 5 min, [95°C for 30 sec, 60°C for 1 min] x 40 cycles, 4°C for 5 min, 90°C for 5 min, 10°C to store.

Analysis of the results. We used the QuantaSoft software (BioRad) with manual thresholding. Good distribution of droplets has two clear droplet populations - negative and positive (Figure SM1A). The ‘rain’ effect is an increased number of droplets with intermediate fluorescence (Figure SM1B). Note that we had a bad batch of PCR plates that lead to shifts of detected fluorescence within a single reaction (Figure SM1C).

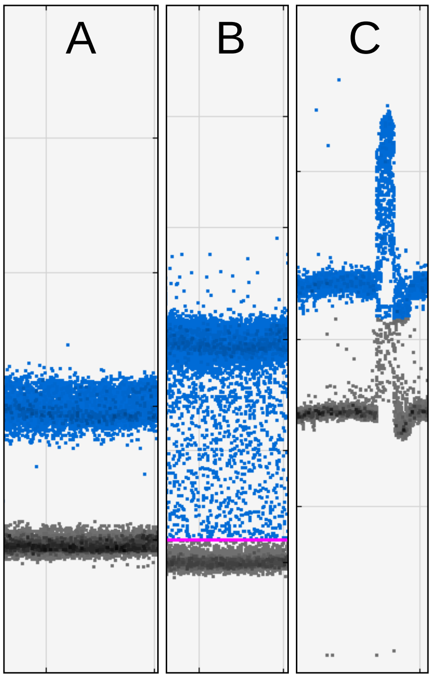

Figure SM1. *ddPCR droplet readout*

A, Normal droplet distribution. ***B***, “Rain” droplet distribution (for example, due to poor primer efficiency or inappropriate handling of the generated droplets). ***C***, Shifted droplet distribution due to bad plate batch.

1. Kang,Q., Parkin,B., Giraldez,M.D. and Tewari,M. (2016) Mutant DNA quantification by digital PCR can be confounded by heating during DNA fragmentation. *Biotechniques*, **60**, 175–185.

2. Yuryev,A. (2007) PCR Primer Design. *Sci. Justice - SCI JUSTICE*, **47**, 173.

3. SantaLucia,J. (1998) A unified view of polymer, dumbbell, and oligonucleotide DNA nearest-neighbor thermodynamics. *Proc. Natl. Acad. Sci. U. S. A.*, **95**, 1460–5.
